# Effect of Src Kinase inhibition on Cytochrome c, Smac/DIABLO and Apoptosis Inducing Factor (AIF) Following Cerebral Hypoxia-Ischemia in Newborn Piglets

**DOI:** 10.1038/s41598-017-16983-1

**Published:** 2017-11-30

**Authors:** Panagiotis Kratimenos, Ioannis Koutroulis, Beamon Agarwal, Stamatios Theocharis, Maria Delivoria-Papadopoulos

**Affiliations:** 1 Department of Pediatrics, Division of Neonatology, Children’s National Medical Center, The George Washington University, School of Medicine and Health Sciences, Washington, DC USA; 2 Department of Pediatrics, Division of Emergency Medicine, Children’s National Medical Center, The George Washington University, School of Medicine and Health Sciences, Washington, DC USA; 3 First Department of Pathology, National and Kapodistrian University of Athens, School of Medicine, Athens, Greece; 4 Department of Hematopathology, Montefiore Medical Center, Bronx, NY USA; 5 Department of Pediatrics, Drexel University College of Medicine, Philadelphia, PA USA

## Abstract

We have previously shown that cerebral Hypoxia-ischemia (HI) results in activation of Src kinase in the newborn piglet brain. We investigated the regulatory mechanism by which the pre-apoptotic proteins translocate from mitochondria to the cytosol during HI through the Src kinase. Newborn piglets were divided into 3 groups (n = 5/group): normoxic (Nx), HI and HI pre-treated with Src kinase inhibitor PP2 (PP2 + HI). Brain tissue HI was verified by neuropathological analysis and by Adenosine Triphosphate (ATP) and Phosphocreatine (PCr) levels. We used western blots, immunohistochemistry, H&E and biochemical enzyme assays to determine the role of Src kinase on mitochondrial membrane apoptotic protein trafficking. HI resulted in decreased ATP and PCr levels, neuropathological changes and increased levels of cytochrome c, Smac/DIABLO and AIF in the cytosol while their levels were decreased in mitochondria compared to Nx. PP2 decreased the cytosolic levels of pre-apoptotic proteins, attenuated the neuropathological changes and apoptosis and decreased the HI-induced increased activity of caspase-3. Our data suggest that Src kinase may represent a potential target that could interrupt the enzymatic activation of the caspase dependent cell death pathway.

## Introduction

Prolonged cerebral HI leads to a state of primary energy failure, characterized by the depletion of cellular reserves of high energy compounds such as adenosine triphosphate (ATP) and phosphocreatine (PCr), as well as by mitochondrial dysfunction. Electrochemical disruption of the neuronal membrane results in lipid peroxidation, prolonged “open” state of the N-methyl-D-aspartate (NMDA) receptor and a subsequent influx of calcium into the cytosol with the formation of free radicals^1–4^. In addition, calcium influx triggers the formation of focal adhesions through the deactivation of cytosolic phosphatases and activation of protein complexes that extend from the membrane to the nucleus and mitochondria^5,6^. In the cytosolic compartment, FAs consist of several proteins that contribute to cell signaling, motility and migration^7–9^, such as Src kinase, which enters the nucleus by mediating phosphorylation of Calmodulin and Calcium/Calmodulin Kinase IV (CaM kinase IV)^7^. The end result is an increased activation of caspases that lead to DNA fragmentation and cell death^6,10,11^.

HI disrupts the mitochondrial membrane, with leakage of mitochondrial pre-apoptotic proteins into the cytosol, including the Second mitochondria-derived activator of caspase/direct inhibitor of apoptosis-binding protein with low pI (Smac/DIABLO) and cytochrome c and Apoptosis Inducing Factor (AIF)^12,13^. Smac/DIABLO is a small-sized protein (27 kDa) stored in the mitochondria and released into the cytosol as a mature protein along with cytochrome c, maintaining a dynamic protein-protein interaction^14^. Cytochrome c (12 kDa), an important element of the electron transport chain in mitochondria is also involved in the initiation of apoptosis^15^. Cytochrome c is released into the cytosol and binds to the apoptotic protease activating factor-1 (Apaf-1)^16^. Smac is released concurrently with cytochrome c from mitochondria into the cytosol during apoptosis and re-activates the initiator and effector caspases by deactivating the inhibitor of apoptosis protein (IAP) mediated inhibition^17^. It has been demonstrated that this inhibition of IAPs allows for increased caspase activity, leading to cell death. Under physiological conditions, IAP is bound to caspases 3,7 and 9 resulting in their deactivation^15^. Apoptosis-inducing factor (AIF) is released upon the loss of mitochondrial membrane potential and translocates to the nucleus resulting in cell death in a caspase-independent pathway^18^ by binding to the death receptor^19^. It has been previously demonstrated that HI results in increased expression of AIF in the cerebral cortex^20,21^.

Since the only clinically validated treatment for HI encephalopathy in term neonates is hypothermia but with only limited improvement in clinical outcomes, there is a need for the discovery of new signaling pathways for the development of therapeutic targets. In the term neonate affected by HI encephalopathy, the main areas of the brain affected are the hippocampus, sensory and motor cortex, basal ganglia, thalamus, and brain stem^22^. The HI insult may disrupt the formation of central motor pathways and alter the developmental plasticity of the newborn brain resulting in significant long term disabilities^23^.

Our previous work has indicated that HI activates the enzyme Src kinase^24–26^. In the present study we investigated the regulatory mechanism by which the pre-apoptotic proteins translocate from mitochondria to the cytosol during HI through the Src kinase in the cerebral cortex of HI newborn piglets.

## Results

### High Energy Phosphates

In order to ensure energy failure and significant HI, the levels of high energy compounds (ATP and PCr) were measured (μmoles/gr brain tissue). The ATP and PCr levels are shown in Table 1. The HI groups with and without PP2 were similar in the levels of ATP and PCr indicating that they were comparable in their degree of HI. Administration of Src kinase inhibitor (PP2) prior to HI did not restore ATP and PCr levels.

**Table 1:** Levels of high energy phosphates in the cerebral cortex of newborn piglets.

Study Groups	ATP (μmoles/g brain)	Phosphocreatine (μmoles/g brain)
Nx (n = 5)	4.41 ± 0.83	3.39 ± 0.55
HI (n = 5)	2.94 ± 0.67*	1.12 ± 0.27*
PP2 + HI (n = 5)	2.81 ± 0.80*	1.70 ± 0.84*

### Neuropathology

**Figure 1:**
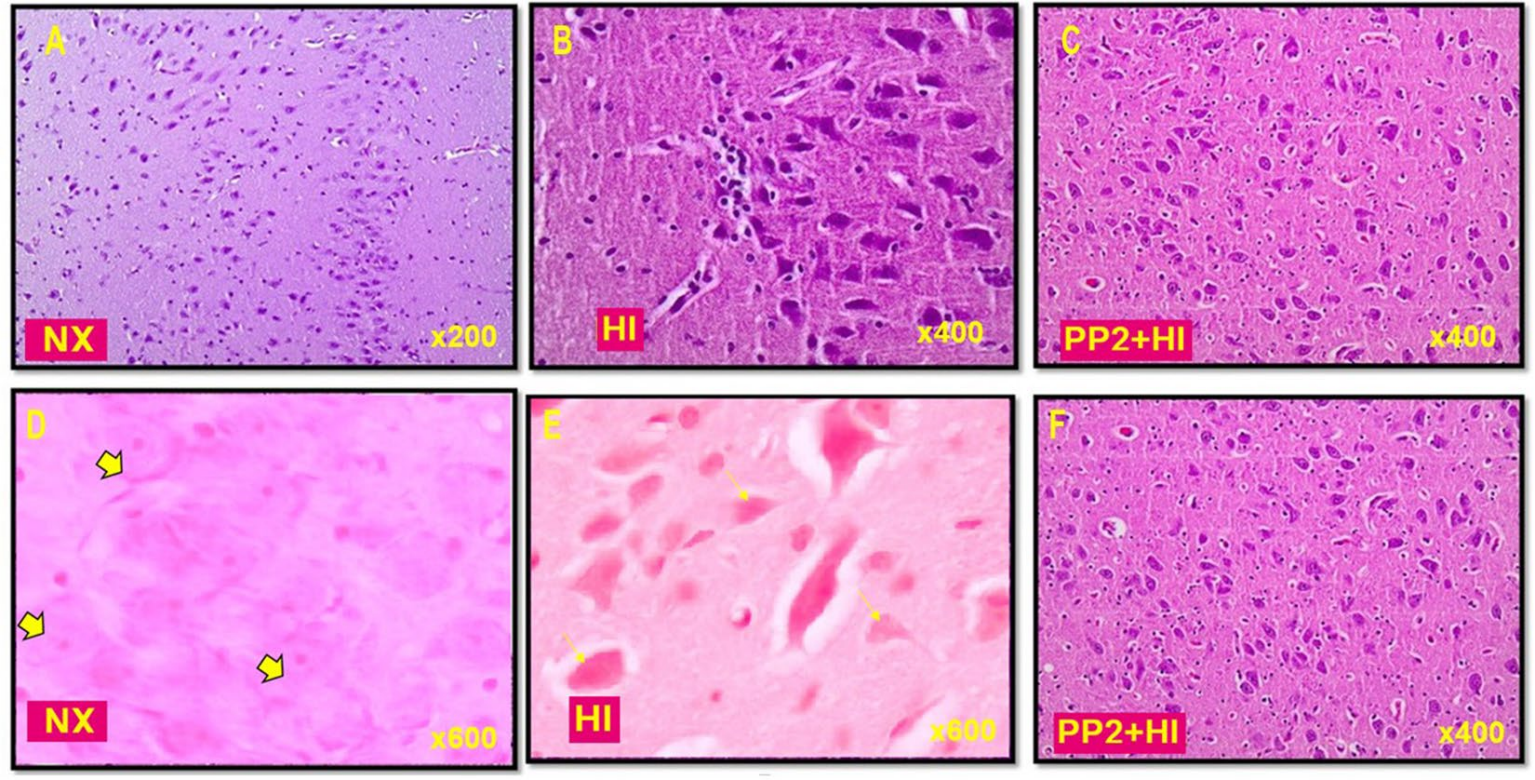
Representative images of H&E histologic staining of hippocampus and putamen from piglets with HI and 2 hours of reoxygenation as well as normoxic controls (n = 5/group). Morphologically normal neurons are shown (**A** and **D**) at the CA1 area of hippocampus. The arrowheads identify normal neurons. Hippocampal HI neurons (CA1 area) are depicted in the middle panels (**B** and **E)**. The arrows denote examples of neurons with classic HI cell injury. The right panels (**C** and **F**) show areas of putamen of HI piglets that were treated with PP2. Photos were taken at ×200 and ×600 on the left, ×400 and ×600 in the middle panel and ×200 on the right panel.

The neuropathology mean score (M ± SD) was 0.33 ± 0.33 in Nx (p < 0.05 vs HI), 3.41 ± 0.34 in HI (p < 0.05 vs Nx) and 2.32 ± 0.23 in PP2 + HI (p < 0.05 vs HI). HI resulted in damaged neurons, pyknotic nuclei, ruptured or irregular bordered nuclear membranes and marked presence of apoptotic profiles. There was also pericellular edema and clustered inflammatory cells. However, Src kinase inhibition resulted in fewer damaged neurons, less karyorrhexis and pericellular edema, and thus attenuated neuronal cell injury compared to the group exposed to HI without Src kinase inhibition Fig. 1.

### Mitochondrial Apoptotic Proteins

**Figure 2:**
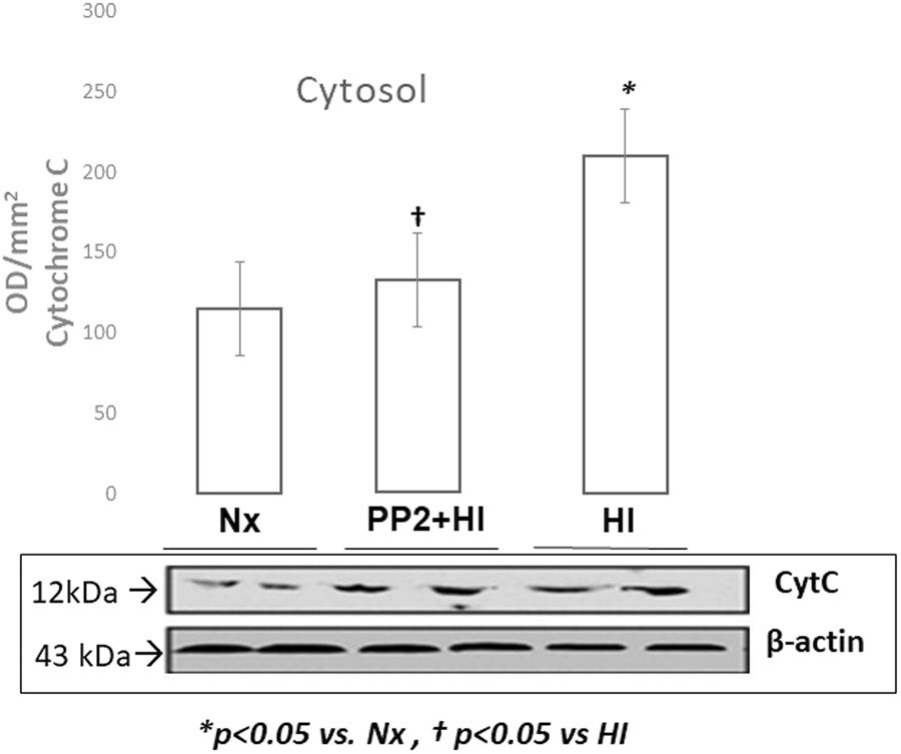
Representative cropped western blot image of cytochrome c levels expressed as optic density(OD/mm^2^) in the cytosol of the cerebral cortex of newborn piglets following 1 hour of HI and 2 hours of reoxygenation with and without pretreatment with PP2 (n = 5/group). HI resulted in increased expression of cytochrome c in the cytosol while pre-treatment with Src kinase inhibitor attenuated its expression.

**Figure 3:**
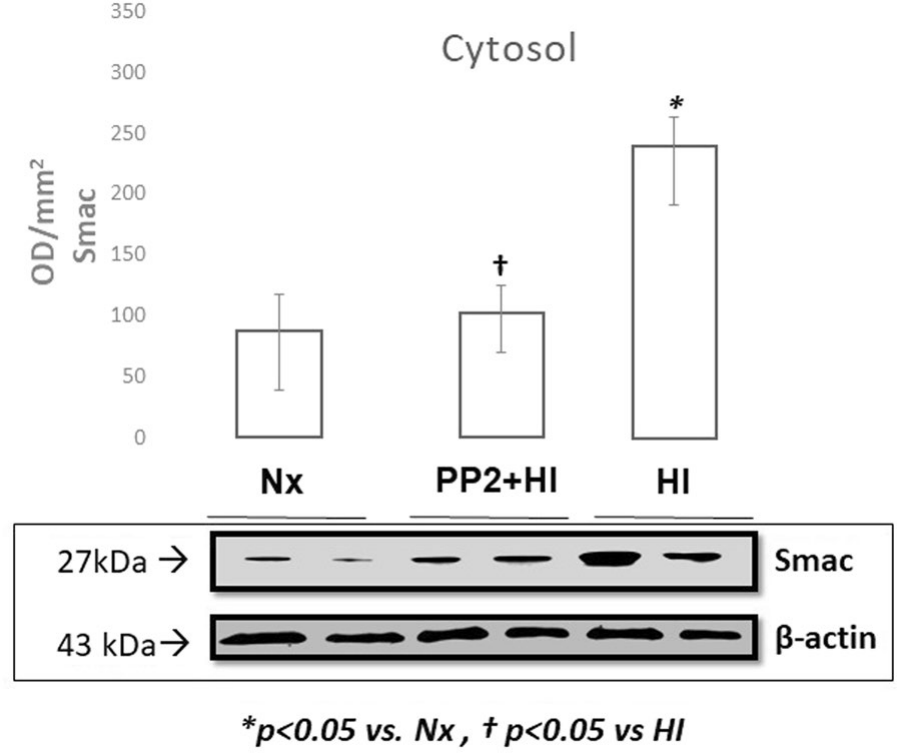
Representative cropped western blot image of Smac/DIABLO levels expressed as optic density (OD/mm^2^) in the cytosol of the cerebral cortex of newborn piglets following 1 hour of HI and 2 hours of reoxygenation with and without pretreatment with PP2 (n = 5/group). HI resulted in increased expression of Smac/DIABLO in the cytosol while pre-treatment with PP2 attenuated its expression.

**Figure 4:**
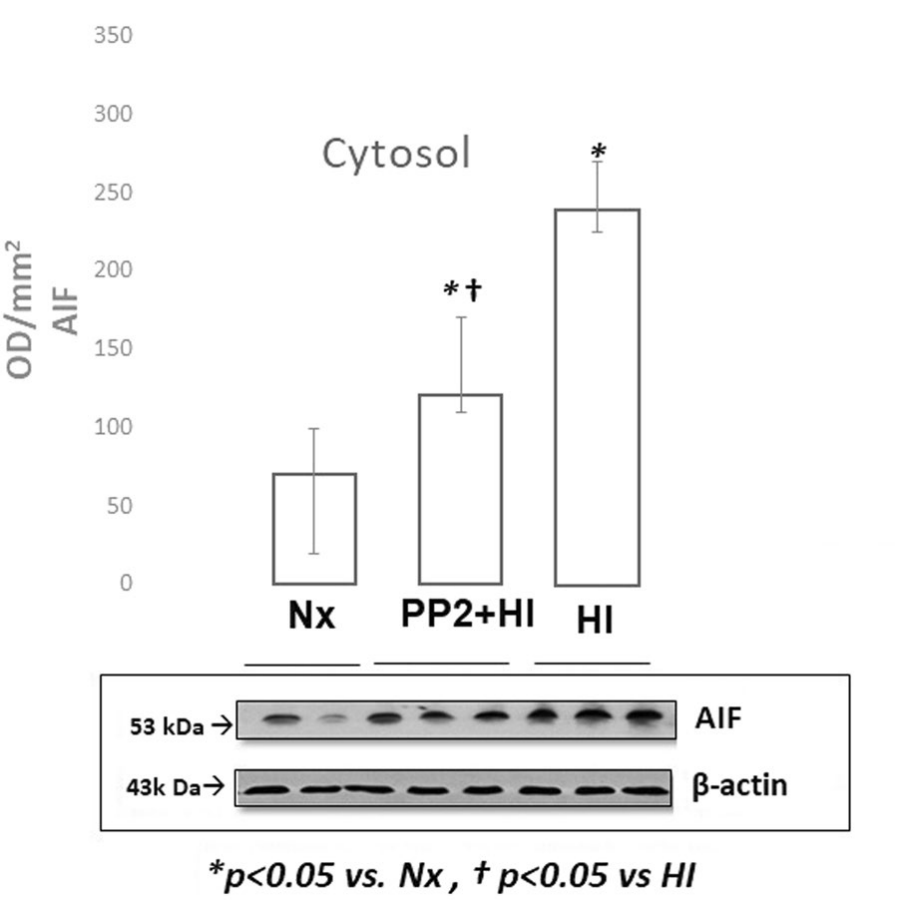
Representative cropped western blot image of AIF levels expressed as optic density (OD/mm^2^) in the cytosol of the cerebral cortex of newborn piglets following 1 hour of HI and 2 hours of reoxygenation with and without pretreatment with PP2 (n = 5/group). HI resulted in increased expression of AIF in the cytosol while pre-treatment with PP2 attenuated its expression.

In order to demonstrate the “leakage” of pre-apoptotic proteins from mitochondria to cytosol we measured the levels of cytochrome c [Fig. 2], Smac/DIABLO [Fig. 3] and AIF [Fig. 4] in both the cytosol and mitochondria [Fig. 5]. In mitochondria, compared to cytosol, the expression of cytochrome c, smac/DIABLO and AIF was increased during Nx and decreased during HI indicating a possible translocation of those proteins during HI from the mitochondria into the cytosol. However, inhibition of Src kinase did not result in any significant changes in the expression of the proteins inside the mitochondria. The results suggest that Src kinase has a regulatory role in preventing the “leakage” of the mitochondrial proteins into the cytosol. In the cytosolic compartment, cytochrome c, Smac and AIF expression was increased following HI compared to Nx (*p* < *0.05)*. However, administration of PP2 attenuated the HI-induced increased expression of each protein (*PP2* + *HI vs HI, p* < *0.05; PP2* + *HI vs Nx, p* = *NS)*.

**Figuree 5:**
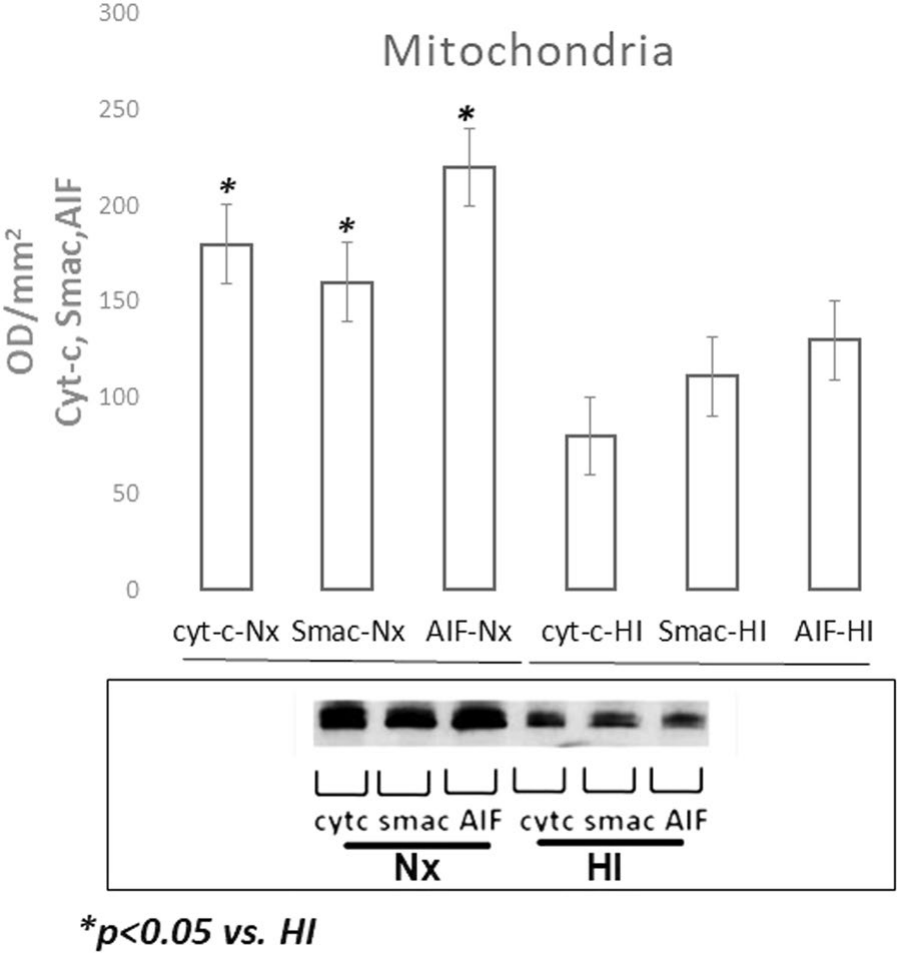
Representative cropped western blot image of cytochrome c, Smac and AIF levels expressed as optic density(OD/mm^2^) in the mitochondria of the cerebral cortex of newborn piglets (n = 5/group) following 1 hour of HI and 2 hours of reoxygenation normalized to the mitochondrial control protein VDAC1 (porin). Following HI, the level of the apoptotic proteins decreased in the mitochondrial fraction while increased significantly in the cytosolic fraction indicating possible translocation from the mitochondria to the cytosol. HI resulted in increased expression of Smac, cytochrome c and AIF in the cytosol.

### Caspase-3 expression and activation

**Figure 6:**
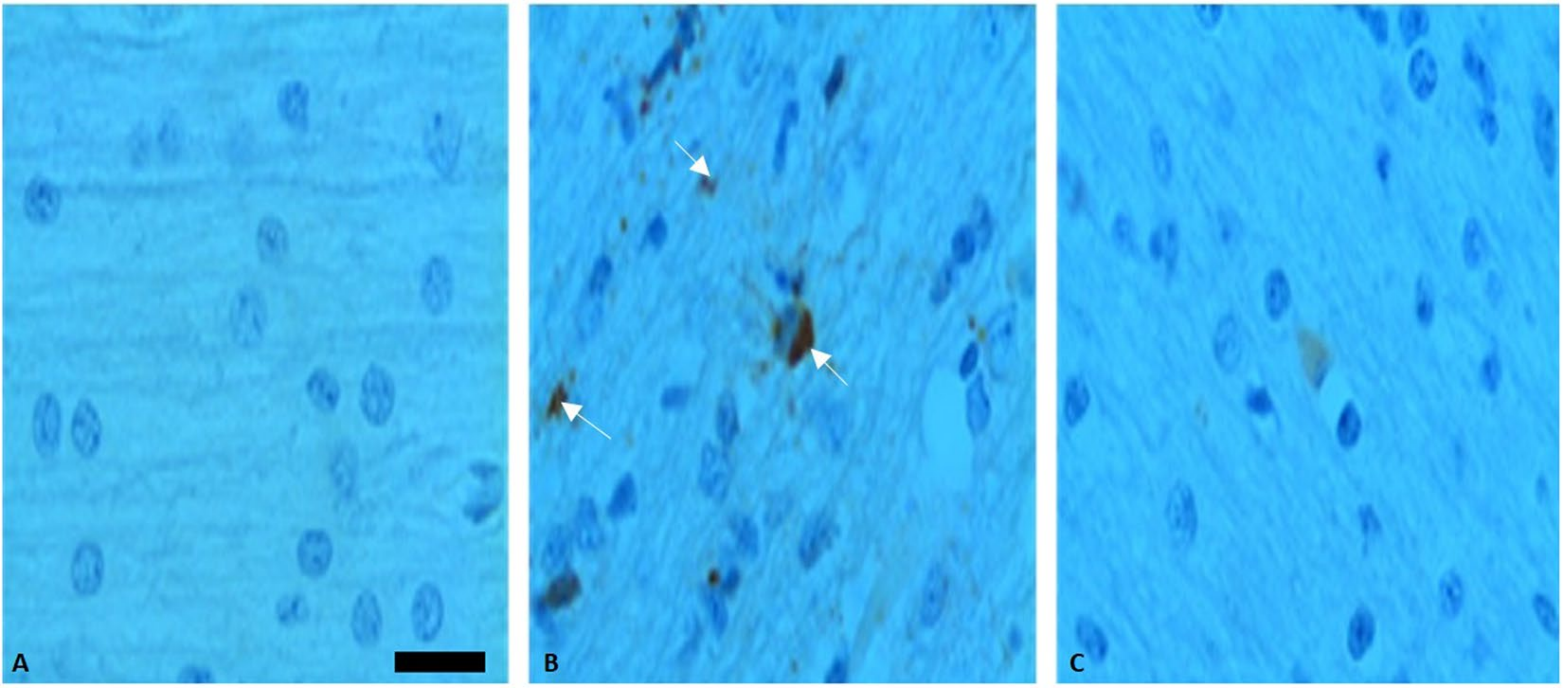
Representative Caspase-3 immunostaining following Src Kinase Inhibition during HI in the cerebral cortex of newborn piglets. There is hardly any positive staining for caspase-3 in Nx (Panel 6-A). HI is characterized by increased caspase-3 expression. The increased cytoplasmic and occasionally membranous staining is pointed by the arrows (Panel 6-B). However, Src kinase inhibition resulted in attenuation of caspase-3 expression (Panel 6-C). Scale bar =100 μm.

**Figure 7:**
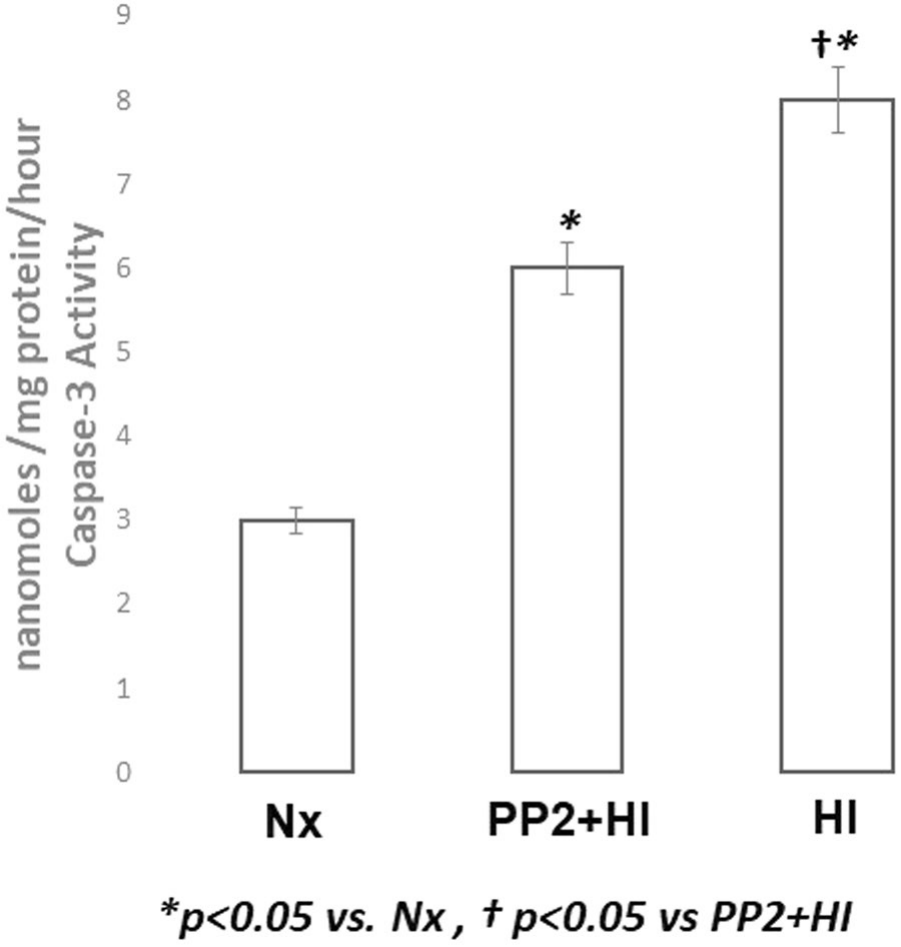
Caspase-3 activity in the cytosolic fraction of the cerebral cortex of normoxic, HI and HI pre-treated with PP2 groups of newborn piglets (n = 5/group). HI resulted in increased activity of Caspase-3 in the cytosol. Pre-treatment with PP2 prevented the HI-induced increased activity of caspase-3 indicating that its activation is regulated by Src kinase. The caspase-3 activity is expressed as nmoles/mg protein/hr.

We used immunohistochemistry to assess the expression of caspase-3 [Fig. 6]. Normoxic animals stained negative for caspase-3 (Panel 6-A). HI was characterized by increased expression of caspase-3 (Panel 6-B). However, Src kinase inhibition resulted in attenuation of caspase-3 expression (Panel 6-C). The activity of Caspase-3 in the cytosolic fraction of the cerebral cortex of normoxic, hypoxic and hypoxic with PP2 is shown in Fig. 7. HI resulted in increased activity of Caspase-3 in the cytosolic fraction of the cerebral cortex of newborn piglets. Pretreatment with PP2 prevented the HI-induced increased activity of caspase-3 indicating that its activation is regulated by Src kinase Fig. 7.

## Discussion

The present study aims to assess the effect of Src kinase inhibition on mitochondrial apoptotic proteins and further delineate cellular mechanisms of HI induced cerebral injury in newborn piglets. We showed that HI resulted in decreased levels of apoptotic proteins in the mitochondria while the cytosolic level of those proteins significantly increased. We also showed that HI resulted in translocation of pre-apoptotic proteins from the mitochondria into the cytosol. Inhibition of Src kinase prevented the increased cytosolic levels of cytochrome c, smac and AIF following HI suggesting that Src tyrosine kinase has a regulatory role in the protein trafficking of the mitochondrial membrane. The exact mechanism by which the aforementioned translocation occurs is a subject of current investigation although several theories have already been developed^12^.

In our laboratory we use the newborn piglet model of HI encephalopathy that shares comparative developmental ontogeny with the term newborn human brain. The distribution of brain injury following global HI in piglets is similar to the distribution of injury to human brain^27^. The newborn piglet is a reasonable model of neonatal encephalopathy due to its large size, presence of gyri and sulci, similar white/grey matter ratio and developmental age at term with human brain. Our group and other investigators^27–38^ have used the newborn piglet model as it provides the advantage of real time measurements of physiologic parameters such as arterial blood gases and continuous blood pressure monitoring which allows titration of the FiO2 to achieve a precise and reproducible degree of HI^27,28,33,39^. Studies have shown that mitochondrial outer membrane permeabilization is the predominant form of apoptosis in the immature brain in comparison to the adult^40,41^. Mitochondria are implicated in numerous processes of the developing newborn brain (cellular powerhouse, programmed cell death, inflammation, formation of reactive oxygen species etc)^22,23,42,43^. Understanding of the mitochondria-mediated cell death pathways and their regulation is crucial in order to identify targets and therapies to alleviate injury of the developing brain. Mitochondria are located in the cytosolic compartment and are embedded in the protein complexes of FAs system. The constantly changing morphology of the FAs enzymes leads to the opening of the mPTP and increased (mitochondrial outer membrane permeabilization(MOMP)^13^. Phosphorylation and activation of the Src kinase may promote the α9 dimerization, expand the mPTP, and allow for the leakage of apoptotic proteins into the cytosol. In contrast, when the enzyme is in its inactive form, Src kinase may block the opening of the mPTP, attenuate the swelling of the mitochondrial membrane and prevent the leakage of the mitochondrial proteins into the cytosol^7^. The increased level of AIF in neuronal cytosol is potentially due to the enhanced expression and translocation of the AIF from mitochondria to the cytosol. We therefore propose that Src kinase-mediated increased AIF expression in the cytosol during HI leads to increased DNA fragmentation and results in caspase-independent programmed neuronal cell death. Src kinase, by tyrosine phosphorylation of calmodulin at Tyr99, activates CaM kinase IV pathway and results to a CREB mediated expression of AIF.

The opening of the mitochondrial permeability transition pore (mPTP) through the inner and outer mitochondrial membrane, allows solutes, water and ions to enter the mitochondrial matrix^12,13^. The Bcl-2 family proteins may be either pro-apoptotic (Bax, BAD, Bak or Bok) or anti-apoptotic (Bcl-2, Bcl-xL, and Bcl-w). Specifically, activated Bax and Bak form pores at the outer mitochondrial membrane in giant unilamellar vesicles allowing transfer of larger molecular weight proteins such as Smac and cytochrome c^44^.

**Figure 8:**
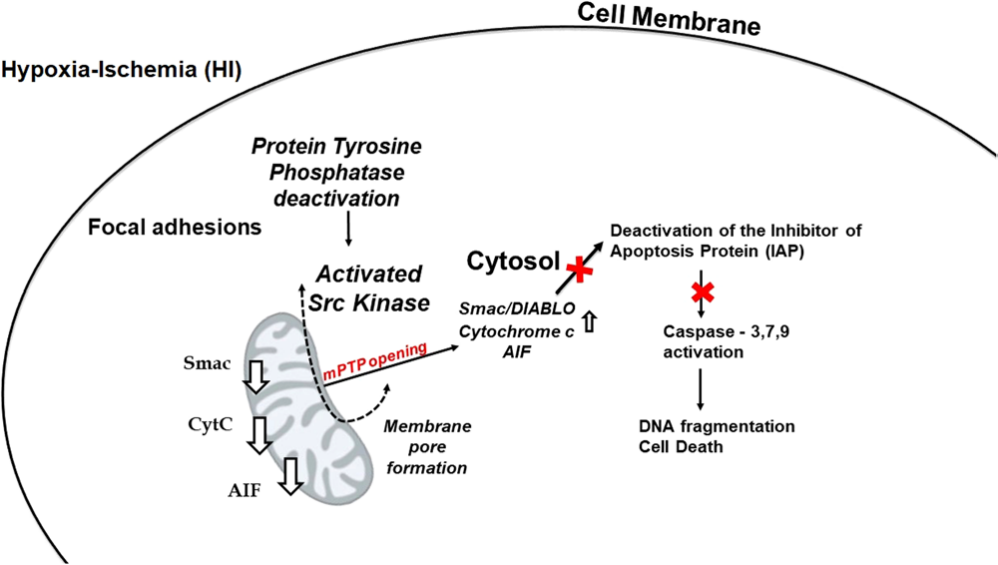
Proposed mechanism by which HI results in the leakage of the mitochondrial pre-apoptogenic agents in the developing brain. HI results in the activation of Src kinase which may open the mPTP resulting in the leakage of the apoptotic proteins into the cytosol. Subsequently, the cytochrome c, Smac and AIF lead to deactivation of inhibitor of apoptosis protein (IAP) which under normal conditions inhibits the activation of caspases 7 and 9. Thus, the caspases 7 and 9 become activated leading to cleavage of the executioner caspase-3, DNA fragmentation and cell death. The figure was created by Dr. Kratimenos.

Protein tyrosine kinases, such as Src kinase, provide a multitude of intracellular signaling pathways including those related to cell survival and programmed cell death^45^. We have previously shown that Src kinase activation during HI occurs via an integrated feedback mechanism, possibly involving other kinases residing in the lipid membrane^46,47^. One of the fundamental initiating events in HI brain injury is the generation of nitric oxide (NO), free radicals and other reactive oxygen species that damage cell membranes, organelles, and even mitochondria^46^. Neuronal nitric oxide synthase (nNOS) is known to decrease the activity of phosphatases and would therefore promote the activation of kinases such as the Src kinase. This activation may further trigger caspase-mediated programmed cell death via the release of mitochondrial protein in the HI newborn brain^16^, which in turn, can further activate caspase-independent pathways through AIF^48^. We propose that HI results in the activation of focal adhesion proteins such as Src kinase which regulates the leakage of the apoptogenic factors from the mitochondria into the cytosol. Subsequently, the cytochrome c, Smac and AIF lead to the activation of caspases -7,-9 and -3 which will result in DNA fragmentation and cell death Fig. 8.

In summary, the current study provides new insights into the regulation of trafficking of the mitochondrial apoptogenic factors by Src kinase. We showed that the HI-induced translocation of apoptotic proteins from the mitochondria into the cytosol is attenuated following inhibition of Src kinase. Together with our previous studies, these data further elucidate the effects of Src kinase on the apoptotic cascade, and identify Src kinase as a potential target to interrupt cell death pathways.

## Materials and Methods

### Experimental Procedures

The experimental animal protocol was approved by the Institutional Animal Care and Use Committee of Drexel University (IACUC Approval Number: 200491). All methods were performed in accordance with the relevant guidelines and regulations. Animal experimentation and induction of HI studies were performed on Yorkshire piglets (Willow Glenn Farm in Strasburg, PA) on day of life 2 or 3. Anesthesia was used for all the procedures described below and all efforts were made to minimize pain and suffering. Newborn piglets were randomly divided into 3 groups (n = 5/group): normoxic, HI and HI pre-treated with PP2. The animals were anesthetized with Isoflurane (4%) and oxygen through a nostril cone and IV access was obtained within 5–10 minutes. Maintenance anesthesia was induced with Isoflurane 1.5%, Fentanyl 50 µg/kg, Pancuronium: 0.3 mg/kg followed by oral endotracheal intubation. For the animals in HI + PP2 group, the PP2 was administered (1 mg/kg, IV) 30 minutes prior to HI in order to attenuate *in vivo* the Src activation in piglets^49^. The animals were ventilated for 1 hour under either normoxic (FiO2 = 0.21) or HI conditions; the later achieved by gradually lowering the FiO2 to 0.06 over 10 minutes. After 1 hour at FiO2 of 0.06, the HI piglets were subsequently re-oxygenated to Nx for two additional hours. Deep anesthesia was maintained throughout HI, recovery and brain harvesting. After the re-oxygenation period, the animals were either transcardially perfused with phosphate buffer saline (PBS) and paraformaldehyde for neuropathology studies or the brain was harvested and placed in liquid nitrogen at −80 °C for biochemical studies. There was a different process that was followed based on whether the animal would be used for biochemical or histopathology studies. For biochemical studies, at the conclusion of each experiment the cerebral cortex was harvested within five seconds following decapitation with a guillotine and a rapid midsagittal craniotomy with “scooping” of the brain parenchyma which was placed in liquid nitrogen. Then the brain was stored at −80° Celsius for subsequent biochemical analysis.

The piglets that were utilized for histopathology analysis, were perfused with paraformaldehyde and then decapitated. The brain was carefully dissected and placed on ice and then, areas of motor and sensory cortex, hippocampus, putamen and caudate nucleus were dissected, prepared and fixed.

### Neuropathology

HI injury was confirmed histologically, by Hematoxylin and Eosin (H&E) stain and evaluation. Upon brain removal, mid-sagittal sections comprising of cerebrum, brainstem and cerebellum en block were fixed immediately by immersion in 10% neutral formalin, processed according to standard neurohistological procedures, and embedded in paraffin. Parasagittal sections corresponding to hippocampus were stained with H&E. Neuropathology was assessed by two independent pathologists and scores ranged from 0 (normal) to 4 (injured neurons >75% of high power field) as described by Hoque *et al*.^50^. Briefly, sections from motor cortex, somatosensory cortex, putamen, hippocampus, caudate nucleus and thalamus were used to evaluate for viable neurons, apoptotic profiles, pericellular edema, pyknotic nuclei and vascular thickening. Cell counting was performed by two different investigators blinded to treatment and clinical course in two specific areas identified using a pig brain atlas namely the CA1 region of ventral hippocampus and the putamen^51^. Morphological cell counting was carried out under light microscopy at 600× magnification using oil immersion lens. In the hippocampus, cells were counted in an ocular field centered on the pyramidal layer of sector CA1 and extending into the stratum oriens and stratum radiatum. In each animal, viable neurons were counted in 8 neighboring, non-overlapping ocular fields along the CA1 region. In the putamen, viable neurons were counted in 8 ocular fields selected randomly in a raster pattern starting in the dorsolateral region of the putamen and ending in the ventral putamen^50^.

Normal neurons were evaluated and counted using morphological criteria. Neurons with well demarcated round nuclei and containing a nucleolar profile were counted. Features of ischemic injury included nuclear hyperchromasia, nuclear pyknosis, cytoplasmic eosinophilia, cytoplasmic shrinkage, cytoplasmic micro-vacuolation, and cell homogenisation^50^.

### Histopathology and Apoptotic Cell Identification

HI injury was confirmed histologically, by Hematoxylin and Eosin (H&E) stain and the caspase-3 expression was determined by immuno-staining. Upon brain removal, mid-sagittal sections comprising brainstem and cerebellum en block were fixed immediately by immersion in cold (4 °C) 10% neutral formalin, processed according to standard neurohistological procedures, and embedded in paraffin. Parasagittal sections corresponding to the paravermis were stained with hematoxylin and eosin (H&E) and cresyl violet (CV), and serial (5 μm) adjacent sections, numbered in sequence, were used for immunostaining. Immunohistochemistry was performed on paraffin embedded material, using antibodies directed to caspase-3, using the three-step immunoperoxidase technique (Biocare Medical, Walnut Creek, CA, USA) as previously described^52^. Sections were counterstained either with methyl green or CV stains.

### Biochemical Studies

#### Isolation of the Cytosolic Fraction

Cerebral tissue mitochondrial fraction was isolated by homogenizing one gram of cerebral cortical tissue by a Dounce-type glass homogenizer (seven strokes with pestle clearance 0.15 mm and seven strokes with pestle clearance 0.07 mm) in 30 ml of fresh isolation medium containing 0.32 M sucrose, 1 mM EDTA and 20 mM Tris–HCl buffer, pH 7.1. The homogenate was centrifuged for 3 min at 1500 × g and the resulting supernatant was centrifuged at 15,000 × g for 10 min to provide the crude mitochondrial pellet. The 15,000 × g supernatant was centrifuged at 100,000 × g for 60 min to obtain the cytosolic fraction^53^, and the protein concentration was determined^54^.

#### Western Blot Analysis

Total protein was extracted from cells using cell lysis buffer (0.5% NP-40, 0.5% SDS, 1.5 mM pH 7.4 Tris-HCL, and 15 mM NaCl). Protein samples (20 μg/lane) were electrophoresed, transferred to polyvinylidene difluoride (PVDF) membranes and incubated overnight with primary antibodies against cytochrome c (sc-7159, Santa Cruz Biotechnology, USA), Smac (sc-22766, Santa Cruz Biotechnology, USA), AIF (sc-9416, Santa Cruz Biotechnology, USA) and the mitochondrial loading control protein VDAC1/Porin (ab15895, Abcam). The membranes were treated with goat-anti-rabbit HRP-conjugated^50^ secondary antibodies (Invitrogen Thermo Fisher, Waltham, Massachusetts, USA). The target protein bands were determined using the reagents visualized provided in the ECL +plus kit (GE Healthcare, Piscataway, NJ, USA) and the immunoreactive band intensities were analyzed with ImageJ (National Institutes of Health, Bethesda, MD, USA). The protein expression was quantified as optic density (OD) per mm^2^.

#### Determination of ATP and Phosphocreatine

Cerebral tissue HI was confirmed by determining the levels of high energy phosphates, ATP and PCr as previously described^55,56^. Briefly, frozen cortical tissue was powdered under liquid nitrogen, as previously described in 6% weight by volume perchloric acid^56^. The extract was thawed on ice and centrifuged at 2,000 g for 15 min at 4 °C. The supernatant was neutralized to a pH of 7.6 using 2.23 M K_2_CO_3_/0.5 M triethanolamine/50 mM EDTA buffer and then centrifuged at 2,000 g for 15 min at 4 °C. Supernatant (300 μL) was added to 1 ml of buffer (50 mM triethanolamine, 5 mM MgCl_2_, 2 mM EDTA, 2 mM glucose, pH 7.6) and 20 μL l NADP. The NADP concentration was 10 mg/ml in 50 mM Triethanolamine (TRA)-HCl buffer. Glucose-6- phosphate dehydrogenase (10 μL) was added and the samples were incubated and read after 8 min. Hexokinase (10 μL) was then added, absorbance readings were taken until steady state was reached and ATP concentration was calculated from the increase in absorbance at 340 nm. Next, ADP (20 μL) and creatine kinase (20 μL) were added to the solution. The samples were read every 5 min for 60 min and phosphocreatine concentrations were calculated from the increase in absorbance at 340 nm.

#### Caspase 3 activity

The activity of caspase-3 was determined spectrofluorometrically at 37 °C using specific fluorogenic substrate for caspase-3 (Ac-DEVD-AMC) obtained from Enzo Life Sciences. Continuous recording was performed at excitation 360 nm/emission 435 nm wave lengths. The activity was determined from the initial slope where the rate of reaction is linear and calculated using the amino-methyl-coumarin (AMC) as standard. The enzyme activity was expressed as nmoles/mg protein/hr.

### Statistical Analysis

Data on ATP, PCr, caspase-3 activity and expression, cytochrome c, Smac/DIABLO, AIF expression and neuropathology were analyzed using one way analysis of variance (ANOVA). A *p* value < 0.05 was considered significant. Pair-wise comparisons between the groups utilized Tukey-test. All values are presented as mean ± standard deviation (SD). The mean neuropathology scores were compared using Kruskal-Wallis One Way ANOVA on Ranks.

## Limitations

The study was performed in 2- or 3- day old newborn piglets because of the practical barriers to obtain 1-day-old newborn piglets. It was not deemed feasible to perform all studies (biochemical assays and histopathology) on the same animals. Biochemical studies such as high energy compounds and enzyme assays require deep freezing of brain tissue to −80 °C at the time of harvest. Conversely, transcardial perfusion and tissue fixation is required for neuropathology. To overcome this limitation, we performed the same experimental process on a larger number of animals in each group in order to perform both histopathologic and biochemical studies. While the pretreatment with Src kinase inhibitor prior to HI may not readily translate to the clinical scenario of neonatal HI injury, the aim of the current study is to delineate the effect of Src kinase on the mitochondrial pre-apoptotic proteins. Future work will focus on the effect of such small molecule inhibitors when administered following the HI event.

### Data Availability

The datasets generated during and/or analysed during the current study are available from Dr. Maria Delivoria-Papadopoulos’ Laboratory at Drexel University College of Medicine.
